# Factors associated with *Salmonella* infection in patients with gastrointestinal complaints seeking health care at Regional Hospital in Southern Highland of Tanzania

**DOI:** 10.1186/s12879-020-4849-7

**Published:** 2020-02-12

**Authors:** Fadhili A. Ngogo, Agricola Joachim, Ahmed M. Abade, Susan F. Rumisha, Mucho M. Mizinduko, Mtebe V. Majigo

**Affiliations:** 1 Muhimbili University of Health and Allied Sciences, Dar es Salaam, Tanzania; 2 Field Epidemiology and Laboratory Training Programme, Dar es Salaam, Tanzania; 3 National Institute for Medical Research, Dar es Salaam, Tanzania

**Keywords:** *Salmonella* infection, Salmonellosis, Gastroenteritis, Antimicrobial resistance, Tanzania

## Abstract

**Background:**

Salmonellosis remains an important public health problem globally. The disease is among the leading causes of morbidity and mortality in developing countries that experience poor hygiene and lack of access to clean and safe water. There was an increase in reported cases of Salmonellosis in Njombe Region, Southern Highland of Tanzania between 2015 and 2016 based on clinical diagnosis. Nevertheless, little is known about the factors contributing to the transmission of this disease in the region. This study was conducted to determine the prevalence, antimicrobial susceptibility, and factors associated with *Salmonella* infection among patients who report gastrointestinal complaints.

**Methods:**

A cross-sectional study was conducted from December 2017 to February 2018 among patients with gastrointestinal complaints at Kibena Regional Hospital. Stool samples were submitted for isolation of *Salmonella* spp. Identification was based on conventional biochemical tests and serotyping to differentiate typhoid and non-typhoid *Salmonella* (NTS). Antimicrobial susceptibility was performed using the Kirby-Bauer disc diffusion method. Multivariable logistic regression analysis was performed to examine the factors independently associated with *Salmonella* infection.

**Results:**

The prevalence of *Salmonella* infection among participants with gastrointestinal complaints was 16.5% (95% CI: 12.7–21.1) of them, 83.7, 95% CI: 70.9–91.5 were NTS while 16.3, 95% CI: 8.5–29.0 were Typhoid *Salmonella* species.

All isolates were sensitive to ceftriaxone and ciprofloxacin, whereas 27.8 and 100% were resistant to co-trimoxazole and ampicillin respectively. The odd of *Salmonella* infection was fourfold higher among participants with formal employment (AOR 3.8, 95% CI, 1.53–9.40). Use of water from wells/rivers (AOR 2.2, 95% CI, 1.07–4.45), drinking untreated water (AOR 2.6, 95% CI, 1.21–5.48) and often eating at a restaurant (AOR 3.4, 95% CI, 1.28–8.93) had increased odds of *Salmonella* infection. Likewise, having abdominal pain (AOR 8.5, 95% CI, 1.81–39.78) and diarrhea (AOR 2.3, 95% CI, 1.12–4.68) were independent symptoms that predict *Salmonella* infection.

**Conclusion:**

There is a high prevalence of *Salmonella* infection among people who report gastrointestinal complaints and it is clinically predicated by diarhoea and abdominal pain. Employed participants and those eating at restaurant and drinking unsafe water had higher risk of infection. *Salmonella* spp. causing gastroenteritis has developed resistance to commonly used antibiotics.

## Introduction

*Salmonella* infection remains a major public health concern worldwide [1], gastroenteritis being the most common manifestation [2]. *Salmonella* serovars causing illness in humans are grouped into two*, Salmonella enterica* serovars referred to as typhoidal *Salmonella* and other serovars grouped as non-typhoidal *Salmonella* (NTS). The NTS is more often the cause of gastroenteritis, an invasive disease frequently occurring in developing countries [3, 4].

*Salmonella* is among common food-borne pathogens predominantly found in poultry, eggs, dairy products, and vegetables, acquired directly or indirectly from human or animal excreta [1, 5, 6]. The risk for infection is high in low-income countries due to poor hygiene and lack of access to safe water and food [3, 6, 7]. The NTS infection, accounts for 93.8 million cases of gastroenteritis and 155 thousand deaths per annum globally [2]. Studies conducted in Tanzania among patients with febrile illnesses revealed that *Salmonella* spp. is among the predominant isolate causing either bacteremia or gastroenteritis [8, 9].

The Tanzania Health Management Information System data of 2016 reported 12,055 cases of salmonellosis in the Njombe region. The reported cases were based on clinical diagnosis due to limited laboratory services. Furthermore, resistance to a commonly used antibiotic in our setting for the treatment of *Salmonella* infection has been reported [4, 10, 11]. In line with the inadequacy of laboratory services, the true magnitude of *Salmonella* infection among patients with gastrointestinal symptoms is not known. Likewise, information on the factors contributing to *Salmonella* infection in the region is limited. This cross-sectional study estimated the prevalence of *Salmonella* infection using culture and identified factors contributing for the transmission of the infection. The finding from this study may assist in setting appropriate measures for the prevention and control of salmonellosis.

## Methods

### Study design, setting and population

This was a cross-sectional study approved by the institutional review board of Muhimbili University of Health and Allied Sciences Ref: MU/PGS/SAEC/Vol.X on 1st Nov 2017. The study was conducted between December 2017 and February 2018 at Kibena Regional Hospital located in the Njombe region, Southern Highland zone of Tanzania. Geographically the hospital is located at latitudes -9^o^ 3085′ and the longitude 34^o^ 7716^′.^ The hospital bed capacity is 160; attend approximately 860 outpatients and 240 in-patients monthly. Gastrointestinal Diseases are among the top 10 diseases at Kibena regional hospital.

The study population was patients seeking medical care at the hospital and clinically suspected with gastrointestinal disease. Patients with variable severity of non-bloody diarrhea, abdominal pain, nausea or vomiting were requested to participate. Only clients whom either self, parents or guardians provided written informed consent to participate were enrolled.

### Sample size and sampling procedure

The representative sample size of participants was estimated using Kish Leslie formula Z^2^P(1-P)/ δ^2^, considering 4.5% prevalence (P) of invasive NTS *Salmonella* infection in Tanga, Tanzania [12], standard normal deviation (Z) of 1.96 at 95% confidence and 3% margin of error (δ). The sample size was estimated to be 301 with the inclusion of 10% non-respondent.

Eligible patients were obtained through a systematic random sampling procedure. The first participant of each day was randomly sampled from the first three patients with gastroenteritis. The subsequent participants were enrolled by skipping one. The expected number of patients with clinical features of gastroenteritis per day was around30, and the minimum of participants expected to be enrolled per day was 15. This sampling procedure was performed on daily basis.

### Questionnaire survey and specimen collection

A structured questionnaire was used to gather information on socio-demographic characteristics (sex, age, marital status, residence, occupation, level of education), clinical presentations (e.g fever, abdominal pain, headache, diarrhea, joint pain, body malaise, nausea and cough), and factors that increase the risk of *Salmonella* transmission (source of water, eating place, type of food, history of illness, water treatment, hygiene practices and domestic animals keeping). Patients or guardians were clearly instructed and illustrated on the amount and how to collect stool samples into a clean leak-proof container. After collection, unpreserved specimens were transported to the laboratory for processing within two hours.

### Laboratory procedures

**Fig. 1:**
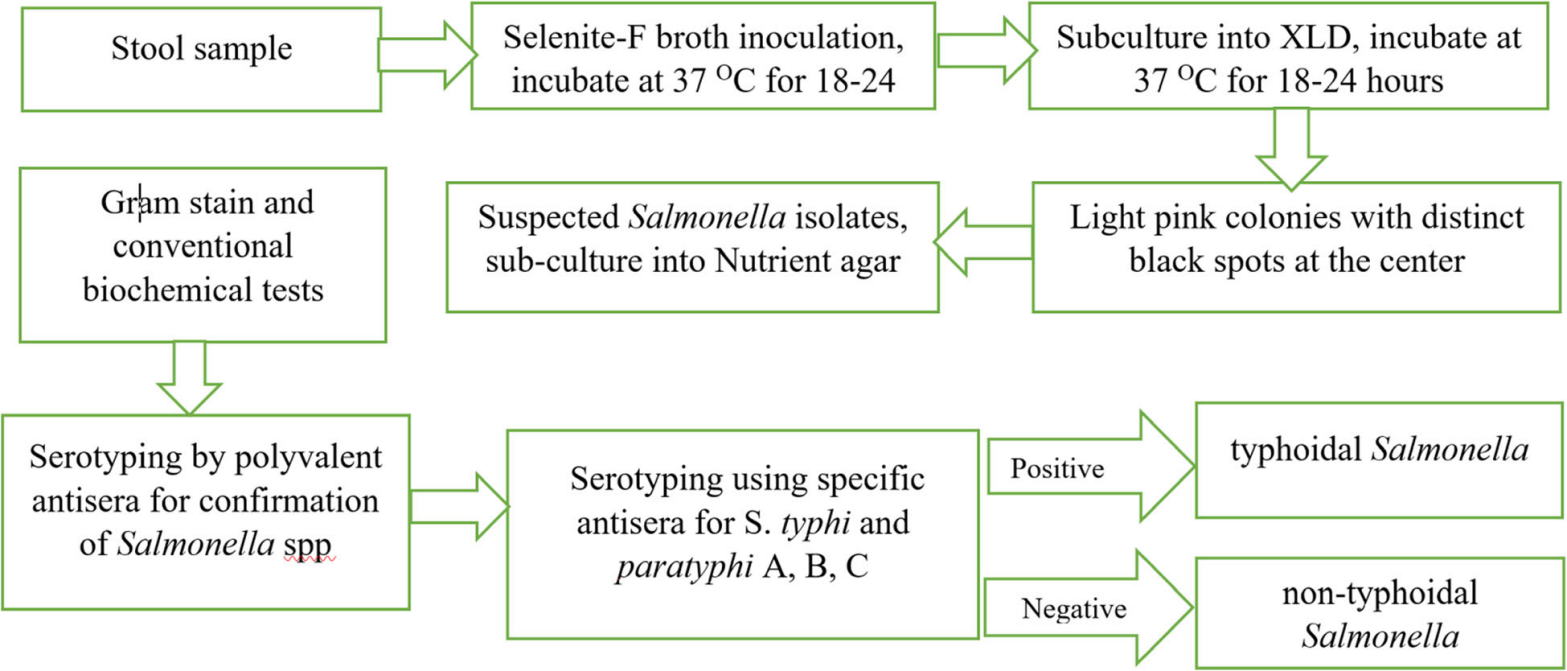
Workflow of laboratory procedure for diagnosis of *Salmonella* spp

Isolation and identification of *Salmonella* species were conducted according to the Global Foodborne Infections Network laboratory protocol [13] and summarized in Fig. 1. About 1–2 g of stool sample was first inoculated into Selenite-F broth (HiMedia Laboratories Ltd., India) and incubated aerobically at 37 °C for 18–24 h. Thereafter, the samples from Selenite F broth was streaked on Xylose lysine deoxycholate agar (Oxoid Ltd., UK) and incubated aerobically at 37 °C for 18–24 h.

The clear to light pink colonies with distinct black spots at the center were considered as *Salmonella* isolates. Suspected colonies were sub-cultured into Nutrient agar (Oxoid Ltd., UK) to get enough pure colonies for further identification. Preliminary identification was based on Gram stain reaction and conventional biochemical tests including triple sugar iron and sulfide indole motility tests (Oxoid Ltd., UK). Identified *Salmonella* spp. were confirmed by using agglutination reaction to polyvalent antisera (Denka Seiken, Tokyo Japan). Serotyping was performed by using specific antisera for S. *typhi* and *paratyphi* A, B, C (Biocan diagnostic, Canada), a nonreactive salmonella spp. were considered NTS (Fig. 1).

Antimicrobial susceptibility testing (AST) was performed using Kirby-Bauer’s disc diffusion method [14]. Briefly, standard inoculums were prepared by direct colony suspension in normal saline, compared with 0.5 McFarland turbidity standards and inoculated on Muller-Hinton agar (Oxoid, UK). The plates with a maximum of six disks were incubated at 35 ± 2 °C for 16–18 h at the inverted position. The results were interpreted according to Clinical and Laboratory Standards Institute guidelines [14]. *Escherichia coli* ATCC 25922 was used as a reference organism for quality control. The antibiotic disks included for AST were: ceftriaxone (30 μg), ciprofloxacin (5 μg), chloramphenicol (30 μg), ampicillin (10 μg) and trimethoprim-sulphamethoxazole (1.25/23.75 μg) (Oxoid Ltd., UK). Selected antibiotic disks for AST were commonly used in our setting for the treatment of salmonellosis.

### Data management and analysis

Statistical Package for Social Sciences version 20 was used for all data analyses. Descriptive analysis for categorical variables was summarized in the form of frequencies and percentages, while continuous variables were summarized as mean ± Standard deviation. Socio-demographic characteristics, clinical presentations, factors that increase the risk of *Salmonella* transmission were independent variables while salmonella infection was dependent variable. The prevalence of *Salmonella* infection was calculated as proportion of positive stool culture among all cultures and expressed in percentages. Comparisons between the groups were done using Chi-Square test.

The Univariate logistic regression analysis was conducted to estimate the factors associated with *Salmonella* infection. The crude odds ratio (cOR) of each independent variable was determined. Variables with *p-value* ≤ 0.2 in univariate analysis were considered in multivariable logistic regression analysis to examine the associations between the S*almonella* infection and independent variables after adjustment for other variables. Associations in the multivariable logistic models were presented as adjusted odds ratio (AOR) estimated at 95% confidence intervals. Interaction between independent variables were examined and the Wald test was used to test the associations of the variables. The Hosmer-Lemeshow test was used to examine the overall fitness of the model. The level of significance was specified at 0.05.

## Results

### Socio-demographic characteristics of study participants

A total of 297 participants were enrolled in the study, the majority, 173 (58.2%) were female. The mean age of participants was 26.7 years ±16.3 standard deviation. Age group 20 to 44 years 148 (49.8%) had the most respondents. Participants with primary education (40.1%) constituted the majority as well as unemployed (39.7%) and single marital status (52.9%). (Table 1).

**Table 1:** Socio-demographic characteristics of study participants

Variables	Frequency	Percentage (%)
Sex		
Male	124	41.8
Female	173	58.2
Age group (years)		
1–12	48	16.2
13–19	58	19.5
20–44	148	49.8
≥ 45	43	14.5
Occupation		
Business	29	9.8
Employed	50	16.8
Farming	100	33.7
None	118	39.7
Level of education		
None	27	9.1
Primary Education	119	40.1
Secondary	91	30.6
Higher Education	60	20.2
Marital status		
Divorced	2	0.7
Married	132	44.4
Single	157	52.9
Widowed/widower	6	2.0

### Clinical presentation of study participants

**Fig. 2:**
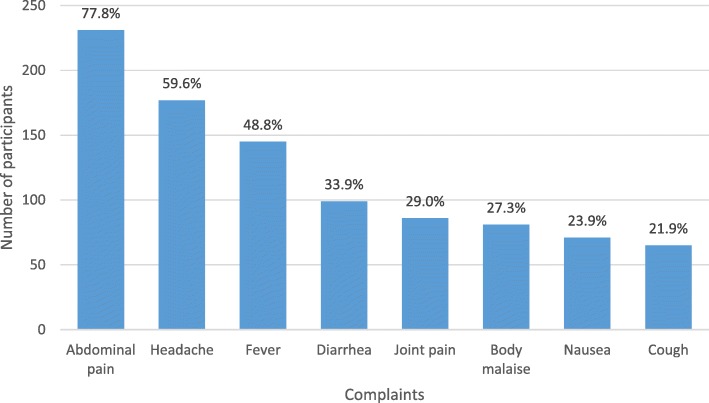
Clinical presentations of participants enrolled in the study. The percentages of participants with each complaint are indicated at the top of the bars

Most participants presented with a history of abdominal pain (77.8%), followed by headache (59.6%) and fever (48.8%). Only 99 (33.7%) presented with Diarrhoea (Fig. 2).

### Prevalence of *Salmonella* infection

Out of 297 stool cultures, 49 (16.5, 95% CI: 12.7–21.1) were positive for *Salmonella,* of which 41 (83.7, 95% CI: 70.9–91.5) were NTS, while eight (16.3, 95% CI: 8.5–29.0) were Typhoid *Salmonella* species. Among the typhoid species, five were *Salmonella typhi* and three were *Salmonella paratyphi*. There was no significant difference in the proportion of *Salmonella* infection between females (19.1, 95% CI: 13.9–25.6) and males (12.9, 95% CI: 8.1–19.9). The lowest prevalence of *Salmonella* infection (8.3%) was observed in children between 1 and 12 years, while the prevalence was almost similar ranging from 16 to 19% in age groups above 12 years. Employed participants had the highest proportion of *Salmonella* infection (30.0, 95% CI: 19.1–43-7) compared to other occupations (13.7 95% CI: 10.0–18.7) (Table 2). There was no significant difference in proportion of *Salmonella* isolation rate among participants with formal education (16.3, 95% CI 12.4–21.2) and informal education (18.5, 95% CI 8.2–36.7) as well as among married participants (18.9, 95% CI: 13.2–26.5) and unmarried (14.5, 95% CI 10.0–20.7) (Table 2).

**Table 2:** Proportion of *Salmonella* infection among participants with socio-demographic characteristics and clinical presentations

Variables	Total	Number of Positive	Proportion positive (95% CI)	*P* -value
Overall	297	49	16.5 (12.7–21.1)	
Sex				
Male	124	16	12.9 (8.1–19.9)	0.158
Female	173	33	19.1 (13.9–25.6)	
Age group (years)				
1–12	48	4	8.3 (3.3–19.5)	0.615
13–19	58	10	17.2 (9.6–28.9)	
20–44	148	28	18.9 (13.4–26.0)	
≥ 45	43	7	16.3 (8.1–30.0)	
Occupation				
Employed	50	15	30.0 (19.1–43.7)	**0.005**
Others	247	34	13.8 (10.0–18.7)	
Education				
Informal	27	5	18.5 (8.2–36,7)	0.767
Formal	270	44	16.3 (12.4–21.2)	
Marital status				
Married	132	25	18.9 (13.2–26.5)	0.311
Unmarried	165	24	14.5 (10.0–20.7)	
Abdominal Pain				
Yes	231	47	20.3 (15.7–26.0)	**0.001**
No	66	2	3.0 (0.8–10.4)	
Fever				
Yes	145	26	17.9 (12.5–25.0)	0.516
No	152	23	15.1 (10.3–21.7)	
Headache				
Yes	177	27	15.3 (10.7–21.3)	0.483
No	120	22	18.3 (12.4–26.2)	
Diarrhea				
Yes	99	25	25. 3 (17.7–34.6)	**0.004**
No	198	24	12.1 (8.3–17.4)	
Joint pain				
Yes	86	20	23.3 (15.6–33.2)	**0.06**
No	211	29	13.7 (9.7–19.0)	

*Salmonella* infection was more prevalent in patients presenting with abdominal pain (20.3, 95% CI: 15.7–26.0 vs. 3.0, 95% CI, 0.8–10.4, *p* < 0.05) and diarrhea (25.3, 95% CI: 17.7–34.6 vs. 12.1, 95% CI: 8.3–17.4). There was no significant difference in the proportion of *Salmonella* infection between participants with and without fever, joint pain and headache (Table 2).

Patients using water from wells or rivers had a higher proportion of *Salmonella* infection (24.4, 95% CI: 16.7–34.2) than those using tape water (13.0, 95% CI: 9.1–18.3), *p* < 0.05. *Salmonella* infection was more prevalent in the sampled individuals with preference of drinking untreated water (22.2, 95% CI: 16.4–29.2 vs. 10.1, 95% CI: 6.1–16.2, *p* = 0.005) and eating at restaurants (27.9 95% CI: 16.8–42.7 vs. 14.6, 95% CI: 10.8–19.4, *p* = 0.029). There was no significant difference in proportion of *Salmonella* infection with or without eating stewed beef, drinking raw milk, keep animals at home, hands washing in basin and preference of salad (Table 3).

**Table 3:** Proportion of *Salmonella* infection with participants’ behavior and practices

Variable	Total	Number of Positive	Prevalence (%)	(95% CI)	*P*-value
Source of water					
Wells/River	90	22	24.4	16.7–34.2	**0.015**
Tape water	207	27	13.0	9.1–18.3	
Drink untreated water					
No	158	35	22.2	16.4–29.2	0.005
Yes	139	14	10.1	6.1–16.2	
keep animal					
Yes	131	23	17.6	12.0–24.9	0.679
No	164	26	15.9	11.1–22.2	
Eat at restaurants					
Yes	43	12	27.9	16.8–42.7	0.029
No	254	37	14.6	10.8–19.4	
Hand wash practice					
On the Basin	115	23	20.0	13.7–28.2	0.196
Running water	182	26	14.3	9.9–20.1	
Eat salad					
Yes	133	18	13.5	8.7–20.4	0.215
No	164	31	18.9	13.6–25.6	
Eat stewed beef					
Yes	67	13	19.4	11.7–30.4	0.467
No	230	36	15.7	11.5–20.9	
Drink raw milk					
Yes	5	2	40.0	11.7–76,9	0.153
No	292	47	16.1	12.3–20.7	

### Antimicrobial susceptibility pattern

Antimicrobial susceptibility was performed on 36 out of 49 (73.5%) isolates. Amongst the isolate assessed for antimicrobial susceptibility, 8(100%,) and 28(68.3%) were typhoid *Salmonella* and NTS respectively. ST All isolates were sensitive to ceftriaxone and ciprofloxacin. Resistance to ampicillin, tetracycline, co-trimoxazole and chloramphenicol were 100, 75.0, 27.8 and 16.7% respectively. Resistance were observed more in typhoid *Salmonella* species than NTS for tetracycline (88% vs 71%) and co-trimoxazole (100% vs 7%). Out of 36 isolates, 9 (25%) were multidrug resistance (MDR) being resistant to 3 or more groups of antibiotics (Table 4).

**Table 4:** Antimicrobial susceptibility pattern of *Salmonella* isolates from participants with gastrointestinal complaints (*N* = 36)

Name of Antimicrobial	Susceptible isolates *N* (%)	Intermediate resistance isolates *N* (%)	Resistant isolates *N* (%)
Ceftriaxone	36 (100.0)	0 (0.0)	0 (0.0)
Ciprofloxacin	36 (100.0)	0 (0.0)	0 (0.0)
Chloramphenicol	28 (77.8)	2 (5.6)	6 (16.7)
Co-trimoxazole	25 (69.4)	1 (2.8)	10 (27.8)
Tetracycline	7 (19.4)	2 (5.6)	27 (75.0)
Ampicillin	0 (0.0)	0 (0.0)	36 (100.0)
MDR			9 (25.0)

### Factors associated with *Salmonella* infection

The odds of having *Salmonella* infection was eight times among patients reported abdominal pain (AOR 8.5, 95% CI, 1.81–39.78) as compared to patients without abdominal pain. Having diarrhea had two times odds of having *Salmonella* infection (AOR 2.2, 95% CI 1.13–4.24) compared to patients without diarrhea. The odd of having *Salmonella* infection were three times among employed participants (AOR 3.03, 95% CI 1.42–6.49) than other occupations. (Table 5).

**Table 5:** Association of *Salmonella* infection with socio-demographic characteristics, clinical symptoms, and behavioral factors among participants with gastrointestinal complaints

Variables	COR (95% CI)	*p*-value	AOR (95% CI)	*p*-value
Male (Ref: Female)	0.6 (0.33–1.20)	0.158	0.5 (0.23–1.09)	0.083
Employed (Ref: Unemployed)	2.7 (1.33–5.43)	**0.005**	3.03 (1.42–6.49)	**0.004**
Abdominal Pain	8.2 (1.93–34.62)	**0.001**	8.5 (1.81–39.78)	**0.007**
Diarrhea	2.5 (1.31–4.57)	**0.004**	2.19 (1.13–4.24)	**0.020**
Joint pain	1.9 (1.01–3.59)	**0.045**	1.8 (0.97–3.77)	0.060
Wells/River Source of water (Ref: Tape water)	2.2 (1.15–4.04)	**0.015**	2.02 (1.05–3.88)	**0.035**
Drink untreated water	2.5 (1.30–4.95)	**0.005**	2.6 (1.21–5.48)	**0.014**
Eat at restaurant	2.3 (1.07–4.82)	**0.029**	3.4 (1.28–8.93)	**0.032**
Wash hand in basin (Ref: Running water)	1.5 (0.81–2.78)	0.196	1.3 (0.60–2.61)	0.545

Key: *AOR* Adjusted odds ratio, *CI* Confidence interval, *COR* Crude odds ratio, *Ref* Reference

The odd of having *Salmonella* infection was three times more among participants regularly eating at restaurants (AOR 3.4, 95% CI 1.28–8.93). Participants who reported using water from rivers or well had two times probability of *Salmonella* infection compared to those using tape water (AOR 2.02, 95% CI 1.05–3.88), whereas drinking untreated water had three times higher chances of having *Salmonella* infection compared to those who drink treated water (AOR 2.6, 95% CI 1.21–5.48) (Table 5). There were no potential interactions between wells/river source of water and drink untreated water as well as employment and eating at restaurant on *Salmonella* infection HIV status was checked, the interaction term was not significant. The Hosmer-Lemeshow test result was *p =* 0.52 which indicated the fitness of the overall model.

## Discussion

The current study has observed a considerable high prevalence of *Salmonella* infection among patients with gastrointestinal complaints. Some factors with significant contribution to *Salmonella* infections among people reporting gastrointestinal symptoms attending regional hospital in the Njombe region were identified. Study participants who reported regular eating at restaurants, use of water from the river or well and drinking untreated water had two to three times risk of having a *Salmonella* infection. This study has also demonstrated that *Salmonella* infection was found more among participants with employment, such that among enrolled individuals, employed had nearly four times more likelihood of *Salmonella* infection than non-employed.

The study found abdominal pain and diarrhea being the clinical determinants of *Salmonella* infection. Although abdominal pain, headache, and fever were the most predominant clinical symptoms similar to reports from other studies [3, 15], headache and fever had an insignificant association with the detection of *Salmonella* in stool culture. The findings of this study is comparable to reports from Kenya [4] and Ethiopia [5] where diarrhea was associated with salmonellosis.

The prevalence of *Salmonella* infection obtained in this study was higher than the findings from some studies in Africa reporting on *Salmonella* infection [4, 5, 9]. On the other hand, our finding was lower than the study conducted in Kenya [6]. The variation between studies on *Salmonella* infection could be due to differences in the type of patients enrolled such as age, immune status or underlying conditions, predisposing factors, as well as variations in bacteriological techniques and specimen used. In some studies, like the present one, only stool culture was performed, while other studies performed blood and stool cultures. For example, the study in Ethiopia [5] included all patients presenting with diarrhea while our study considered those attending clinician suspected gastroenteritis.

The prevalence of *Salmonella* infections varies from region to region depending on social-economic status, water sanitation, and hygiene practices [12, 16]. The availability of clean and safe water has been a key determinant of the prevalence of *Salmonella* infection [6]. Therefore, the variations in different regions for clean, safe and scarcity of water could explain the variations in magnitude and contributing factors for *Salmonella* infection. The study found that drinking untreated water particularly from rivers or wells among people with gastrointestinal complaints had a high likelihood of being infected with *Salmonella*. The possible reason is that the river and/or wells are more likely to be contaminated with stool as a result of open defecation or by sharing with animals.

The present study observed the increased risk of *Salmonella* infection among employed people, who reported gastrointestinal complaints than unemployed. We speculate people with employment frequently eat at restaurants during working hours which increased the risk of infection. The association of *Salmonella* infection with eating in restaurant is an indication that some restaurants do not observe proper hygienic conditions for food preparation and storage, hence increasing the likelihood of *Salmonella* infection [3, 6].

The study found all *Salmonella* isolates sensitive to ceftriaxone and ciprofloxacin comparable to a study conducted in Kenya [17]. It should be noted that resistance of *Salmonella* spp. to fluoroquinolones has been reported in Uganda and Japan [18, 19]. Sadly, high level of resistance towards ampicillin (100%) and tetracycline (75.0%); moderate resistance to co-trimoxazole (27.8%) and chloramphenicol (16.7%) were observed. The findings are in accord with to the study done in Kenya [4] with some difference to ampicillin (27.9%). The emergence of multidrug-resistant (MDR) among *Salmonella* spp. has been reported in some African countries such as Kenya [4] and Ethiopia [5]. The current study also demonstrated the presence of MDR *Salmonella* spp. considering the level of resistance to ampicillin, tetracycline, co-trimoxazole, and chloramphenicol. The high level of resistance could be attributed partly by the irrational use of antibiotics in our settings as previously reported [6, 20]. In general, these results call for continuous monitoring of antimicrobials stewardship.

Blood culture is also considered the most suitable diagnostic method for *Salmonella* infection especially typhoid fever [3]. However, the technique is very expensive, requiring a well-equipped laboratory and not readily available in many health facilities in developing countries. One of the limitation of this study was that blood culture was not performed. This might have affected the isolation of typhoid *Salmonella* spp. thus underestimate the overall prevalence of Salmonellosis in this study. Due to limited resource, this study only differentiated typhoid and NTS without performing specific serotyping for NTS. The study is hospital based among sick suspected cases hence the findings could not be inferred to the general population.

## Conclusions

There is a high prevalence of *Salmonella* infection among people who report gastrointestinal complaints and it is clinically predicated by diarhoea and abdominal pain. Employed participants and those eating at restaurant and drinking unsafe water had higher risk of infection. Our finding call for health management teams to ensure continued public health education on hygienic practices. There is a need for regular antimicrobial testing to control and prevent salmonellosis with multidrug-resistant *Salmonella* spp.

## Data Availability

The datasets used during the current study are available from the corresponding author on a reasonable request.

## Abbreviations

AOR: Adjusted odds ratio
AST: Antimicrobial susceptibility testing,
CI: Confidence interval
COR: Crude odds ratio
MDR: Multidrug-resistant
NTS: Non-typhoidal *Salmonella,*
